# Unveiling the Influence of the Curve of Spee on Bite Force and Chewing Ability: A Comparative Study

**DOI:** 10.1155/2024/6533841

**Published:** 2024-02-21

**Authors:** Zainab Ali Alkhalaf, Mohammed Ghazi Sghaireen, Kiran Kumar Ganji, Mohammad Khursheed Alam, Rakhi Issrani, Raghad Mohammed Alsaleh, Sultana Zamil Almegren, Mahmoud Gamal Salloum

**Affiliations:** ^1^Department of Prosthetic Dental Sciences, College of Dentistry, Jouf University, Sakaka, Aljouf, Saudi Arabia; ^2^Department of Preventive Dentistry, College of Dentistry, Jouf University, Sakaka, Aljouf, Saudi Arabia; ^3^Department of Periodontology & Oral Implantology, Sharad Pawar Dental College, Datta Meghe Institute of Higher Education & Research, Sawangi (Meghe), Wardha, India; ^4^Department of Research Analytics, Saveetha Dental College and Hospitals, Saveetha Institute of Medical and Technical Sciences, Saveetha University, Chennai, India; ^5^Center for Transdisciplinary Research (CFTR), Saveetha Dental College, Saveetha Institute of Medical and Technical Sciences, Saveetha University, Chennai, India; ^6^Department of Public Health, Faculty of Allied Health Sciences, Daffodil lnternational University, Dhaka, Bangladesh; ^7^Department of Periodontology and Oral Implantology, Ministry of Health, Dental Clinics Complex West of Riyadh, Laban, Riyadh, Saudi Arabia; ^8^Department of Substitutive Dental Sciences, College of Dentistry & Pharmacy, Buraydah Private Colleges, Buraydah, Saudi Arabia

## Abstract

**Aim:**

To investigate the impact of the curve of Spee (CS) accentuation on bite force, chewing ability, and additionally, other factors that influence chewing ability and bite force such as restorations, caries, gender, habits, and TMJ problems.

**Materials and Methods:**

231 participants (118 male and 113 female, mean age = ±27.96 years) were recruited for this cross-section study. Participants completed a data collection sheet in which age, gender, Angle's classification of malocclusion, overjet, overbite, TMJ problems, habits, restorations, and caries experience were recorded. Two examiners made a lower impression, chewing ability test and measured the bite force for each participant. Measurement of the CS was obtained digitally from the poured dental cast, and the categorization was as follows: flat (<1 mm), normal (1-2 mm), or deep (>2 mm).

**Results:**

The mean maximum bite force (MBF) was 468.722 N for participants with flat CS, 389.822 N for normal CS, 647.08 N for deep CS, and 384.667 N for reverse CS. The average MBF was higher for participants with deep CS (*p* < 0.05). MBF force was higher in males. However, BMI was not significantly related to MBF values. Participants with normal and flat CS have comparable chewing capacity (*p* > 0.05). Also, a significant difference in bite force and chewing ability was found between the three categories of CS (*p* < 0.05).

**Conclusion:**

Bite force variations across various CS types were linked to gender and habits. Chewing ability showed no differences concerning gender, habits, TMJ problems, caries, or restorations, emphasizing CS's significant impact on bite force while showcasing the unchanged nature of chewing ability amidst diverse factors.

## 1. Introduction

The human jaws are complex structures that serve a variety of purposes. They are an important component of the stomatognathic system because they chew and break down food before transferring it to the alimentary canal. Maximizing masticatory effectiveness while minimizing stress on the masticatory system and teeth is one of the primary objectives of all dental treatments [1]. Muscles of mastication work efficiently when their action is in harmony with the alignment of teeth. Detentions are arranged according to different curves: the curve of Monson, Wilson, and Spee [2]. The curve of Spee (CS) is a significant component that contributes to establishing the natural occlusion. It allows for defining the normal functional protrusive movement of the mandible. It has been previously discussed how the CS affects force distribution in the oral cavity and how modifications to the CSCS are necessary after extensive orthodontic and prosthetic therapy [3, 4]. The removal of stresses from the condyle, as well as the maxilla and mandible, has been said to be essential for improving masticatory efficiency and lowering total strain on a patient's oral cavity [5].

There was a notion that CS served a biomechanical purpose in food processing by enhancing the crush-shear ratio between the posterior teeth and the effectiveness of the occlusal force. The forward inclination of the posterior teeth increased the crush surfaces of the molars, according to Osborn and Bragar 1987. They related the depth of the CS to the bite force by a mathematical model. They concluded that as the average occlusal plane is raised from 6 to 0 below the parallel plane passing through the condyles, the sum of the muscle tensions required to produce a given force along the axis of any given tooth decreases [6]. Then, Osborn in 1993 continued his investigation to determine if a relationship could be established between the crush-shear force and the sagittal plane of the anterior-posterior teeth. According to his theory, humans' superficial masseter muscle is positioned correctly for crushing food between the posterior molars, which have occlusal surfaces that tilt forward and serve as the best illustration of the CS [7].

Masticatory efficiency, or masticatory ability, refers to the placing of food in the mouth, and the subsequent biting, crushing, and grinding activities that mix it with saliva to form it into a bolus before it is swallowed [8–10]. This complex task is subject to a number of variables such as bite/occlusal forces, mandibular kinetics, occluding teeth, salivary activation, and tongue function. Numerous methods exist for assessing masticatory function. It should be noted that the bite force is a biological variable, which is potentially associated with masticatory efficiency; however, it can be employed as a measure against which proper masticatory function is determined [11, 12]. Essentially, it is the force produced by the activity of the masticatory muscles and the jaw's elevator muscles, which are modified by craniomandibular mechanics [13], temporomandibular dysfunction [14], restorations [15], caries [16], and different stages of periodontitis [17, 18]. Recently, a number of studies on age-related masticatory efficiency have decided there is no direct link between aging and mastication [19]. Moreover, age-related decline in occlusal force, salivary flow, medication, and how many natural teeth a person retains have also been linked to reduced masticatory efficiency [12, 20, 21]. Studies indicated that gender differences in the masticatory function of elderly individuals with complete natural dentition emphasize the importance of considering these distinctions in masticatory function evaluations [22–24].

One of the crucial therapeutic procedures involved in the rehabilitation of numerous long-span posterior restorations concerns the re-establishment of the occlusal plane. This re-establishment aims to maximize masticatory efficiency while minimizing any load on the masticatory system. Indeed, the foundation of the optimal tooth arrangement is dependent on the restoration of compensatory curves, which promotes harmony between the anterior teeth and condylar guidance [25]. However, previous studies did not demonstrate obvious relation between the accentuated and reverse CS and its effect on bite force and chewing ability [5].

Therefore, the aim of the study was to investigate the impact of CS accentuation on bite force, chewing ability, and additionally, other factors that influence chewing ability and bite force such as restorations, malocclusion, caries, gender, habits, and TMJ problems.

## 2. Materials and Methods

### 2.1. Study Population

The cross-sectional study took place at the Department of Prosthodontics, Dental College, Jouf University in Sakaka, Saudi Arabia, spanning from June 2021 to January 2023. Ethical approval was granted by the University's Local Committee of Bioethics (9-04/43). The sample size was calculated using G Power 3.1.9.2 software from Heinrich–Heine–Universität Düsseldorf, Germany, employing a 95% confidence interval, 80% power (*α* = 0.05). Participants were recruited from screening clinics, with enrollment limited to those meeting the specified inclusion criteria. Prior to any examinations, each participant provided informed consent by signing a consent form and received easily comprehensible information about the study's objectives. Participants were assured of the voluntary nature of their involvement and their right to withdraw at any time. Of 751 volunteers screened, 239 met the inclusion criteria, while 5 declined participation, and three individuals were excluded due to a history of peanut allergy.

### 2.2. Participant Characteristics

The inclusion criteria for participation in the research encompass individuals possessing fully erupted upper and lower teeth from the second molar to the central incisor on both the right and left sides, aged 18 or above, and demonstrating mental fitness. Individuals with patients with medical conditions that could affect their chewing ability or a history of previous trauma to the TMJ area, extensive dental restorations, ongoing orthodontic treatment, mixed dentition, noncarious tooth wear, periodontal problems, endodontically treated tooth #6, food allergies related to the study, missing opposing teeth to the lower first molar, and orofacial pain were excluded from the study.

### 2.3. Data Collection

Upon enrollment in the study, participants underwent a comprehensive assessment to gather current medical and dental histories alongside demographic details, including age, gender, education, marital status, and occupation. The primary investigator (ZAK) and the supervisor (MGS) conducted a clinical examination using a dental mirror (15/16 inch, Hahnenkratt, Königsbach-Stein, Germany) and a dental explorer probe (0700-9, anatomical handle single ended; ASA Dental, Bozzano, Italy) in a dental unit. Bitewing X-rays were taken for investigating participants when proximal carious lesions were anticipated on the maxillary and mandibular first molars and for patients displaying grade I mobility to assess periodontal status. Independent variable data such as age, gender, Angle's classification of malocclusion, overjet, overbite, TMJ problems, habits, restorations, and caries experience and the dependent variable data such as bite force and chewing ability were collected using a customized data collection form and transferred onto Microsoft Excel software.

### 2.4. Measurements of CS and Depth

The study casts were positioned in a base former aligning the right posterior teeth parallel to the former's border. After sizing and aligning the occlusal plane, the casts were set on holders to ensure parallelism. Using a Nikon Coolpix S4000 camera at a 90° angle, photographs of the casts were taken and analyzed on a computer. Corel DRAW X5 software, with 800% enlargement, electronically measured the CS from the digital images. Reference planes were established from canine buccal cusps to the distobuccal cusp tips of the second molars, enabling precise measurement using a “dimension tool” to determine perpendicular distances. Perpendicular lines were drawn from this reference line to key dental landmarks, such as the first molar, mesiobuccal cusp of the second molar, and premolar cusp tips, defining the depth of the CS as the greatest length observed [26]. The CS depth categories were based on Lie's previous study: flat (<1 mm), normal (1-2 mm), or deep (>2 mm) [27].

### 2.5. Estimation of Chewing Ability

The study employed three standardized food portions (carrot, apple, and peanut) to assess participants. Carrot and apple were each cut into 1 cm cubes, and the peanuts (averaging 1 g) were utilized. Participants, seated upright without head support, chewed three pieces of each test food. The test foods were randomly distributed using a lottery system to ensure patient anonymity. Five calculations were recorded for the masticatory strokes employed by the participants to chew the food portions, namely, food mastication included time until initial swallow, time until mouth empty, number of masticatory actions before the first swallow, number of masticatory actions until mouth empty, and number of swallows before the mouth was free of food. To prevent muscular fatigue, a 4--5-minute break allowed participants to drink and rinse their mouths. Two observers, situated in front of the participants, recorded time and counted masticatory strokes. The mean values from the three recordings were used to determine chewing ability [28].

### 2.6. Bite Force Registration

Bite force was assessed using GM10 Occlusal Force Meter, a device equipped with a hydraulic pressure gauge and a vinyl biting element, displaying digital Newton units. Participants were familiarized with the equipment and instructed to exert maximum bite force upon the transducer's placement on the lower first molar while seated in an upright position in a dental chair, maintaining the Frankfort plane parallel to the floor. Average left- and right-side bite force values were recorded for participants meeting the inclusion criteria on both sides. Measurements were conducted before the chewing test to prevent muscle fatigue.

To assess reliability, intraexaminer and interexaminer measurements were performed on 10 participants. Intraexaminer reliability, evaluated with an AK value of 0.87, indicated excellent agreement, while interexaminer reliability, determined by a well-trained intern dentist, showed a BK value of approximately 0.86, signifying satisfactory agreement. An independent-sample *t*-test and a MANOVA test were used to compare the significant difference in chewing ability and bite force male for various independent variables.

## 3. Results

In total, 231 (118 males and 113 females) participants were assessed for the outcome variables such as chewing ability and bite force. The age group of the participants ranged from 18 to 54, with a mean age of 27.96 (±7.22). The bite force of the participants ranged from 147.5 to 952.5 N, with a mean bite force of 491.62 (±187.64). The chewing ability of the participants ranged from 3.13 to 17.63, with a mean chewing ability of 8.03 (±2.26) (Tables 1 and 2).

The *t*-test analyses revealed notable findings regarding various parameters in the study population (Table 3). Gender-based comparisons unveiled a significant disparity in bite force between males and females (*t* = 6.95, *p* < 0.05), with males demonstrating substantially higher bite force than females. Conversely, no significant gender-based differences were observed in chewing ability (*t* = −0.90, *p* > 0.05). Evaluation of restorations indicated no substantial impact on either bite force (*t* = 1.29, *p* > 0.05) or chewing ability (*t* = 1.48, *p* > 0.05). Similarly, the presence or absence of TMJ problems did not significantly affect bite force (*t* = 0.78, *p* > 0.05) or chewing ability (*t* = −0.31, *p* > 0.05). However, participants with habits exhibited significantly higher bite force than those without habits (*t* = 10.1, *p* < 0.05), while chewing ability remained unaffected (*t* = 9.9, *p* > 0.05). Surprisingly, the presence of caries did not influence bite force (*t* = 24.19, *p* > 0.05) or chewing ability (*t* = 26.88, *p* > 0.05).

The MANOVA test results revealed no significant differences in bite force among different Angle's classifications of malocclusion (Wilks' lambda = 0.643, *p* > 0.05) (Table 4). The mean bite force values for class I malocclusion, class II division 1, and class II division 2 were 495.645, 462.833, and 469.818, respectively. Similarly, for chewing ability, no significant differences were found among the different malocclusion classifications (Wilks' lambda = 0.332, *p* > 0.05). The mean chewing ability scores for class I malocclusion, class II division 1, and class II division 2 were 7.984, 9.088, and 7.719, respectively. These findings suggest that, in this study, malocclusion classifications did not exert a statistically significant influence on either bite force or chewing ability.

The MANOVA test (Table 5) revealed a significant difference between reverse, flat, normal, and deep CS when considered jointly on the variables bite force and chewing ability (Wilks' lambda =0.00, *p* < 0.01).

Post hoc comparison was conducted using the Tukey test to determine the individual group differences with respect to bite force and chewing ability (Table 6). In case of bite force, there was a statistically significant difference in deep CS in comparison with reverse, flat, and normal CS (*p* < 0.05). In case of chewing ability, there was a statistically significant difference in flat and deep CS (*p* < 0.05).

## 4. Discussion

This study used the GM10 digital bite force transducer in order to accurately assess the participants' bite force. With the ability to measure a bite force of up to 1000 N, the GM10 dynamometer's key advantages are its portability, secure, and comfortable recording method. Furthermore, the dynamometer's accuracy and repeatability functions are well established, having been utilized effectively in a number of other studies to measure human bite force [3, 25, 29]. The bite force was measured on the first molar on both sides for each participant. The first molar was selected due to the larger area and periodontal ligament surrounding the roots of posterior teeth and stronger bite force that can be tolerated. Moreover, different parts of the oral cavity have different ranges of bite force, and the highest bite force was found on the posterior teeth [30]. Contrary to many other studies, we have used five parameters instead of chewing gum or typical sieving techniques to evaluate masticatory function. We did evaluate a variety of natural test foods, though, including soft and hard food. We chose an effective and simple method that counts the number of chewing strokes and the time it takes to prepare the test food for swallowing. This approach did not require the participants to remove the masticated test material or pay particular attention to the food, which is what typically happens when people eat [31]. The test foods were offered to the participants using a predetermined random order system, which helped standardize the methodology. As previously stated that occlusal force and chewing ability decline with tooth loss, only those with entirely natural full dentition were selected for this study [4, 9, 32].

It was found that the bite force mean values for the male and female participants were 585.8 and 397.4, respectively, which was statistically significant (*p* < 0.05). These results corroborate findings from other research, which indicate that males possess significantly higher maximal bite force than females [10, 33, 34]. One explanation for this is that female teeth tend to be smaller, which corresponds to smaller periodontal ligament regions and thus, less powerful bite force. In addition, males' larger muscular potential, which can be explained by anatomical differences between the sexes, is likely to be a further contributing factor [34]. The only research that could be found to refute this was conducted on durophagous species. That study examined whether variations in bite force could be a reflection of the dimorphism in head size and shape of the durophages *Malaclemys terrapin*. The findings of that research showed that the female terrapins, whose heads are larger than their bodies, had a more powerful bite force than the males [35]. However, that study was applied on animal subjects, while the current study was on humans.

In terms of chewing ability, despite the fact that there was a preference for men over women, the differences were insignificant (*p* > 0.05), and this result concurs with previous studies by Kosaka et al. [9] and Ikebe et al. [19]. On the other hand, in 2003, a study by Ono et al. found that the preference for masticatory ability was related to age, gender, and dental condition and that masticatory ability was significantly superior in males. According to Poli et al. [12], the decline in bite force with age does not directly correlate with malnutrition in the elderly, as other factors like natural dentition, frailty, and comorbidities play significant roles in chewing capability. Providing a unique and broader perspective compared to existing literature predominantly focused on young and adult populations. These differences could be explained by the fact that in their study, they used gummy gel as a test food which is influenced by cognitive performance [24, 36]. Another study was undertaken in 2016 that compared masticatory characteristics and the ability to predict masticatory efficiency in male and female adolescents. In that study, the participants were aged 14–17 years old, and the sample size was smaller than in the current study. Nevertheless, the results showed that boys chewed more frequently and possessed greater masticatory efficiency than girls [24]. Manzon et al. [37] indicated that patients using overdenture prostheses experience improved bite force, although not reaching the levels observed in fully dentate subjects. Notably, bite force does not exhibit a correlation with the body mass index despite the increased prevalence of obesity observed in edentulous subjects or those with prostheses.

Another study by Gudipaneni in 2020 also used juveniles to find the effect of all types of dental caries on the bite force of children aged 7–9 years, as it related to their first permanent molars. These results suggest that bite force decreases as the incidences of caries increase [38]. The aforementioned discrepancies in this study may be accounted for by the inclusion of individuals with occlusal proximal caries involving enamel and/or dentin, whereas in the research under discussion, participants with caries that went beyond pit and fissure were not included. Another issue to be considered is the type of prosthetic restorations as reported by the study that the type of removable denture influences bite forces and chewing efficiency, with only CoCr-RPDs objectively restoring satisfying chewing function [39].

The study examined bite force and chewing ability in individuals displaying signs but no symptoms of temporomandibular problems, finding no significant differences between those with or without TMJ issues (*p* > 0.05 for bite force, *p* > 0.05 for chewing ability). Contrasting findings from Shimada et al. indicated that introducing pain into the masseter muscle did not notably impact masticatory efficiency or bite force, aligning with Pizolato's study showing no substantial reduction in maximum bite force in young individuals with TMJ disorders and bruxism [40]. However, research comparing affected and unaffected sides in unilateral TMJ problems revealed markedly reduced bite force in affected areas, contrasting with no significant differences in bite force in bilateral TMJ disorders. Notably, the absence of participants with painful TMJ in the current study's criteria may explain the discrepancies observed in these findings.

This study examined biting and chewing habits like lip and nail biting and gum chewing, revealing that individuals with established habits exhibited higher occlusal bite forces (*p* < 0.05). Nakagawa et al. found similar results, showing improved occlusal force in the elderly after gum chewing exercises [41]. Shirai et al.'s study, employing gum-chewing exercises, mirrored these outcomes across various facial morphologies [42]. However, Castelo et al. discovered reduced bite force linked to thumb sucking in children with mixed dentition, differing from this study in age group, teeth condition, and bite force gauge used [43]. Notably, despite habitual chewing practices, this study found no significant differences in chewing ability, an aspect largely unexplored in existing literature.

This study examined various malocclusions' impact on bite force and chewing ability suggesting no significant differences across malocclusion types (*p* > 0.05). Singh et al. similarly found no substantial differences in occlusal force among different malocclusion groups [44]. However, contrasting views suggest a significant relationship between bite force and malocclusion severity, particularly on the right side. Yet, this study's comparability is limited due to diverse malocclusion severity and age demographics in the samples, differing from the present research, primarily comprising class I occlusions without class III malocclusion instances. Owens et al.'s findings revealed decreased masticatory efficiency in individuals with malocclusions, while conflicting results exist regarding the impact of malocclusion treatment on chewing ability, with some studies suggesting limited improvement posttreatment due to diverse variables in test foods and assessment materials.

In spite of the apparent significance of deep occlusal curvature in chewing ability, other research suggests that this curve must be positioned in a way that absorbs occlusal stresses during maxillomandibular function [45]. The relative position of the condyle within the mandibular fossa was biomechanically linked to the CS. A flatter CS, for instance, is associated with more posteriorly placed condyles, while a steeper CS was observed to have more anteriorly placed condyles. It may be that a more posteriorly oriented condyle within the mandibular fossa is a contributing factor in cases of anterior displacement of the articular disc, which frequently causes TMJ noises [46]. This infers that a flatter CS may be a risk factor when it comes to TMJ sounds, or that this may be the result of the condyle being more posteriorly placed during growth and maturation. The differences in occlusal curvatures and maxillary arch dimensions between people with symptoms of TMJ disorders and those who were asymptomatic have been investigated by Kanavakis and Mehta [47]. The conclusion was that participants with TMJ symptoms had a flatter CS, which indicates that deep occlusal curvatures are not connected to TMJ or muscular pain [47].

In this investigation, it was found that chewing ability improved in individuals with normal or deep curves, suggesting that masticatory effectiveness increases with the deepening of the occlusal curvature. Another study by Fueki et al. [48] examined whether a connection existed between occlusal curvature and the ability of young adults with permanent dentition to mix and comminute food. They concluded that the participants with flatter curves and a big sphere radius of the occlusal curvature were better at mixing and cutting food. In young adults with permanent dentitions, occlusal curves like the CS seemed to be associated with the ability to mix and cut food. This indicates that subjects with strong bite force possess a mild anteroposterior occlusal curvature [48].

### 4.1. Limitations

The study did not examine participants' dietary impact on the CS and occlusion. Diet influences jaw stress and orofacial bone stimulation, vital for typical occlusal curvature. The CS classifications' effect on occlusal interferences was not explored. The limitations of chewing ability tests include potential issues with age of the sample, subjective assessments, and challenges in interpreting results, which may hinder generalizability and sensitivity to changes in chewing function. Further research is needed to gauge the depth of the CS impacting TMJ and investigate its relationship with occlusal interferences. The study aimed to link CS accentuation with masticatory function, recommending broader research on diet's role, older age groups, diverse malocclusions, and facial morphologies to validate these findings and explore occlusal interferences.

## 5. Conclusion

Within the limitations of the study, it can be concluded that bite force exhibited variance across different CS types, demonstrating gender and habitual differences. However, in contrast, chewing ability did not display variations concerning gender, habits, TMJ problems, caries, or restorations. Thus, the study underscores the impactful role of CS accentuation in bite force, elucidating its influence among diverse factors, while revealing the unaltered nature of chewing ability in relation to these assessed variables.

## Figures and Tables

**Table 1:** Distribution of variables in gender, malocclusion, restoration, habits, caries, TMJ problems, and CS among participants (*N* = 231).

Parameters	Variables	*N*
Gender	Male	118
	Female	113

Malocclusion	Class I	198
	Class II division 1	15
	Class II division 2	18

Restoration	Absent	219
	Present	12

Habits	Absent	214
	Present	17

Caries	Absent	202
	Present	29

TMJ problems	Absent	219
	Present	12

CS	Reverse	5
	Flat	58
	Normal	95
	Deep	73

**Table 2:** Descriptive statistics for the variables such as age, overjet, overbite, bite force, and chewing ability among participants (*N* = 231).

Parameters	Minimum	Maximum	Mean	Std. deviation
Age	18.00	54.00	27.96	7.22
Overjet	0.00	10.00	3.04	1.72
Overbite	0.00	9.00	2.89	1.54
Bite force	147.50	952.50	491.62	187.64
Chewing ability	3.13	17.63	8.03	2.26

**Table 3:** Comparison of bite force and chewing ability among variables such as gender, restorations, TMJ problem, habits, and caries.

Variable	Parameters	Gender	*N*(231)	Mean	Std. deviation	Std. Error mean	*t*	*p*
Gender	Bite force	Male	118	585.83	164.58	19.39	6.95	<0.05
		Female	113	397.40	160.71	18.94		
	Chewing ability	Male	118	7.86	2.36	0.27	−0.90	>0.05
		Female	113	8.20	2.16	0.25		

Restorations	Bite force	Absent	213	497.31	189.27	16.41	1.29	>0.05
		Present	18	422.72	158.01	47.64		
	Chewing ability	Absent	213	8.11	2.32	0.20	1.48	>0.05
		Present	18	7.06	0.94	0.28		

TMJ problem	Bite force	Absent	219	494.40	189.32	16.17	0.78	>0.05
		Present	12	437.07	152.03	57.46		
	Chewing ability	Absent	219	8.01	2.26	0.19	−0.31	>0.05
		Present	12	8.29	2.49	0.94		

Habits	Bite force	Absent	214	481.26	183.09	15.81	10.1	<0.05
		Present	17	630.35	202.64	64.08		
	Chewing ability	Absent	214	8.08	2.23	0.19	9.9	>0.05
		Present	17	7.35	2.64	0.83		

Caries	Bite force	Absent	202	495.09	191.20	17.03	24.19	>0.05
		Present	29	467.27	163.24	38.47		
	Chewing ability	Absent	202	8.13	2.32	0.20	26.88	>0.05
		Present	29	7.33	1.70	0.40		

**Table 4:** Comparison of bite force and chewing ability in relation to various classes of malocclusion.

Outcome parameters	Angle's classification of malocclusion	Mean	Std. error	95% confidence interval		Wilks' lambda	*p*
				Lower bound	Upper bound		
Bite force	Class I malocclusion	495.645	16.945	462.145	529.145	0.643	>0.05
	Class II division 1	462.833	62.899	338.487	587.180		
	Class II division 2	469.818	56.894	357.342	582.294		
Chewing ability	Class I malocclusion	7.984	0.203	7.583	8.386		>0.05
	Class II division 1	9.088	0.754	7.597	10.578		
	Class II division 2	7.719	0.682	6.371	9.068		

**Table 5:** Comparison of bite force and chewing ability in relation to different types of CS.

Outcome parameters	CS classified	*N* = 231	Mean	Std. error	95% confidence interval		Wilks' lambda	*p*
					Lower bound	Upper bound		
Bite force	Reverse	5	384.667	88.085	210.518	558.816	0.000	<0.05
	Flat	58	468.722	25.428	418.450	518.995		
	Normal	95	389.822	19.863	350.552	429.092		
	Deep	73	647.087	22.495	602.613	691.561		
Chewing ability	Reverse	5	10.038	1.263	7.540	12.535		<0.05
	Flat	58	8.646	0.365	7.925	9.367		
	Normal	95	8.232	0.285	7.669	8.795		
	Deep	73	7.167	0.323	6.530	7.805		

**Table 6:** Post hoc comparison of bite force and chewing ability in relation to different types of CS.

Outcome parameters	(I) CS	(J) CS	Mean difference (I-J)	Std. error	Sig	95% confidence interval	
						Lower bound	Upper bound
Bite force	Reverse	Flat	−84.05	91.68	0.79	−322.44	154.33
		Normal	−5.15	90.29	1.00	−239.94	229.63
		Deep	−262.42^*∗*^	90.91	0.02	−498.80	−26.03
	Flat	Reverse	84.05	91.68	0.79	−154.33	322.44
		Normal	78.90	32.26	0.07	−4.99	162.79
		Deep	−178.36^*∗*^	33.95	0.00	−266.64	−90.08
	Normal	Reverse	5.15	90.29	1.00	−229.63	239.94
		Flat	−78.90	32.26	0.07	−162.79	4.99
		Deep	−257.26^*∗*^	30.00	0.0	−335.29	−179.23
	Deep	Reverse	262.42^*∗*^	90.91	0.02	26.03	498.80
		Flat	178.36^*∗*^	33.95	0.00	90.08	266.64
		Normal	257.26^*∗*^	30.00	0.00	179.23	335.29

Chewing ability	Reverse	Flat	1.39	1.31	0.71	−2.02	4.81
		Normal	1.80	1.29	0.50	−1.56	5.17
		Deep	2.87	1.30	0.1	−0.51	6.26
	Flat	Reverse	−1.39	1.31	0.71	−4.81	2.02
		Normal	0.41	0.46	0.80	−0.78	1.61
		Deep	1.47^*∗*^	0.48	0.01	0.21	2.74
	Normal	Reverse	−1.80	1.29	0.50	−5.17	1.56
		Flat	−0.41	0.46	0.80	−1.61	0.78
		Deep	1.06	0.43	0.06	−0.05	2.18
	Deep	Reverse	−2.87	1.30	0.12	−6.26	0.51
		Flat	−1.47^*∗*^	0.48	0.01	−2.74	−0.21
		Normal	−1.06	0.43	0.06	−2.18	0.05

^*∗*^The mean difference is significant at the 0.05 level.

## Data Availability

Data will be made available upon request to the corresponding author.
